# Correction:Predicting Coronary Heart Disease Using Risk Factor Categories for a Japanese Urban Population, and Comparison with the Framingham Risk Score: The Suita Study

**DOI:** 10.5551/jat.Er19356

**Published:** 2016-09-01

**Authors:** Kunihiro Nishimura, Tomonori Okamura, Makoto Watanabe, Michikazu Nakai, Misa Takegami, Aya Higashiyama, Yoshihiro Kokubo, Akira Okayama, Yoshihiro Miyamoto

In this article “Predicting Coronary Heart Disease Using Risk Factor Categories for a Japanese Urban Population, and Comparison with the Framingham Risk Score: The Suita Study” by Kunihiro Nishimura, et al., which appeared in JAT 2014, 21: 784-798 are incorrect.Table 1 on page 787 and “Population Characteristics” on page 788 are corrected.

